# On the Entrance of Coal and Other Insoluble Substances from the Intestinal Canal into the Blood

**Published:** 1847-10

**Authors:** 

**Affiliations:** Dorpat


*On the entrance of Coal and other Insoluble Substances from the Intestinal Canal into the Blood.* By Professor OESTERLEN, in Dorpat. Charcoal rubbed down into a fine powder, and mixed with water, was given to several animals, chiefly rabits, for from three to six days. They all swallowed about one ounce. With the exception of blackened fœcal discharges, nothing anormal was observ ed during life, and when killed, the only unusual appearance discovered was the black colour of the intestinal mucous membrane. In all the animals, however, blood taken from the mesenteric vein, the portal vein, as well as the clots found in the right cavities of the heart, in the liver, spleen, and lungs, exhibited on a microscopic examination, minute pieces of charcoal. The size of these varied from 1.300th to 1.60th, and in some instances to 1.42nd of a line. They were less numerous in the kidney and blood of the vena cava. The urine and bile contained none. Other animals fed on Berlin blue, gave exactly the same result. Thus there can be no doubt that solid and insoluble substances, after being received into the stomach may enter the mesenteric veins, and through them the general circulation.—*Zeitschrift fur Rationelle Medizin, from Dub. Med. Press.*

